# Frequency and intensity of alcohol consumption: new evidence from Sweden

**DOI:** 10.1007/s10198-016-0805-2

**Published:** 2016-06-09

**Authors:** Gawain Heckley, Johan Jarl, Ulf-G Gerdtham

**Affiliations:** 1 Health Economics and Management, Institute of Economic Research, Lund University, Box 117, 22100 Lund, Sweden; 2 Health Economics Unit, Department of Clinical Sciences, Lund University, Medicon Village, 223 81 Lund, Sweden; 3 Department of Economics, Lund University, Lund, Sweden

**Keywords:** Alcohol, Demand, Drinking pattern, Binge drinking, I10, I12, I14

## Abstract

There is an increasing body of evidence that the intensity in which alcohol is drunk is of greater concern than the frequency or overall quantity consumed. This paper provides an extensive analysis of the demand for alcohol as measured by total quantity, frequency, and intensity. A unique large sample of cross-sectional data from Sweden 2004–2011 allows reduced-form alcohol demand equations to be estimated for beer, wine, and spirits, split by alcohol drinking pattern (average vs. binge drinkers) and gender. Results find a negative beer excise rate effect for participation and frequency, and positive effect for intensity. The effect was stronger for binge drinkers. Generally, the results also show a positive socioeconomic (income and education) gradient in frequency demand and a negative gradient in the intensity demand. Female wine drinkers show a positive socioeconomic gradient in both frequency and intensity. The findings highlight the complexity of this policy space. Tax increases appear to reduce frequency but raise intensity consumed. The more educated and higher earners drink more in total, but less intensely when they do and this is likely to explain in part why poor health is concentrated amongst lower socioeconomic status individuals.

## Introduction

The demand for alcohol is not just of interest in its own right, but also because alcohol consumption has important and significant societal costs through adverse effects on crime and health, for example [1]. The traditional approach to the economic analysis of alcohol demand has been to consider the demand for total alcohol consumed in a given period [2]. In taking this approach, it is assumed that the frequency (how often an individual drinks) and intensity of consumption (how much an individual drinks at each drinking occasion) have no bearing on the utility an individual receives. Frequency and intensity are in effect treated as perfect substitutes. This simplifies the analytical task but potentially hides important information about how individuals consume alcohol. The pattern by which individuals consume alcohol is an important factor determining health outcomes and risky health behaviors: evidence from the USA shows that binge drinkers are 14 times more likely to drink drive compared with non-binge drinkers [3] and more generally the risk of death from acute alcohol-related illness has been found to linearly increase with frequency but exponentially increase with intensity [4]. How individuals drink has a bearing on the societal costs of alcohol consumption. Evidence from Sweden, noting that binge drinkers are more likely to be heavy drinkers, found that “at least for health care costs, the cost is quite heavily concentrated in the heaviest drinking group” [1]. Understanding how frequency and intensity decisions affect the overall quantity decision will allow greater understanding as to what influences an individual’s drinking behavior. A policy may have no effect on total quantity for example, but may have an effect on the frequency and intensity decisions that would be masked by solely assessing the quantity decision.

While the economic literature on alcohol has in general focused on modeling the quantity of alcohol demanded, a few studies have examined the determinants of frequency of consumption specifically for binge drinkers [5, 6]. Naimi et al. [7] have studied the socioeconomic factors associated with intensive binge drinking, finding age <35 years, male and less education amongst others as key risk factors. However, even fewer studies have examined both the frequency and intensity decisions together. Berggren and Sutton [2] estimated a structural model of alcohol demand where frequency and intensity entered the budget constraint as a multiplicative term. The authors found that for spirit consumption in Sweden, frequency and intensity are indeed simultaneous sub-decisions of the overall quantity decision and that education and income are negatively associated with intensity but have no effect on frequency. Petrie et al. [8] consider an alternate form of the problem, examining the determinants of the intensity frequency ratio. Rather than modeling the budget constraint as Berggren and Sutton [2] and Petrie et al. [8] define a multiplicative quadratic utility function. The results in Petrie et al. [8] are consistent with Berggren and Sutton [2] in that they find a negative relation of the intensity frequency ratio with education. A consequence of the assumed form of the utility function the authors made is that the intensity frequency ratio is related to neither price changes nor income differences. Given the importance of the budget constraint in defining an individual’s choice set, it is undesirable to assume a utility function that yields this result. More recent evidence from Australia has found a negative association between the price of alcohol and the number of days in which alcohol is consumed lightly, yet found no association between price changes and the number of days of high-intensity drinking [9].

While the research by Berggren and Sutton [2] was pioneering in breaking apart the quantity decision into its constituent parts of frequency and intensity, some empirical issues remained. Prices of alcohol were not included (because the data had no time element), which may be a key component of any demand analysis. The data requirements are also quite demanding when estimating, as the authors did, a structural model. A structural model of the form estimated by Berggren and Sutton [2] for frequency and intensity requires instruments for frequency and for intensity. However, the choice of instruments in Berggren and Sutton [2] are debatable. A priori, it is quite hard to think of many variables that predict the frequency decision and not the intensity decision (and vice versa) when so little is known about how these decisions are made.

The literature on the demand for frequency and intensity of alcohol consumption is under-researched even though it has been shown to be important to consider both frequency and intensity separately. We address this evidence gap in this paper by utilizing new data from Sweden that allows the consideration of the determinants of the demand for frequency and intensity across three particular alcohol types: beer, wine, and spirits. We split the analysis by alcohol type because different individuals drink different alcohol types and there may be systematic differences in the characteristics of these individuals. Importantly, this new data also allows the frequency and intensity decisions to be compared between all of those who drink and the subset who are binge drinkers giving new insights into the binge drinking decision. The dataset is large and allows further breakdown by gender, which is useful because there are important biological differences in how much alcohol women and men can tolerate and also preferences may differ in important ways across the genders. This level of detail ensures not only a better level of understanding of the socioeconomic factors related to alcohol drinking patterns but also yields results that should be of greater relevance internationally. The analysis of a female wine binge drinker in Sweden is more likely to yield results of relevance to other female wine binge drinkers from other countries than results not split by alcohol type, gender, and drinking style participation preference. The paper unfolds as follows: Sect. 2 first presents the data material and following this presents the estimation strategy, Sect. 3 reports the results and Sect. 4 discusses the results and concludes.

## Data and methods

### Data material

#### Monitor project survey description

Individual-level micro-data on individuals’ drinking patterns and background characteristics was collected as part of the Monitor project [10]. This is a repeated cross-sectional survey performed by telephone interviews. A drinker is defined as someone who had an alcoholic drink in the last 30 days prior to the interview. A binge drinker, as defined by the Monitor project study, is someone who in the last 30 days has had one or more episodes where the quantity of alcohol drank was at least: one bottle of wine (75 cl), five shots of spirit (25 ml), four cans of strong beer/cider (>3.5 %) or six cans of low alcohol content beer (3.5 %). The same values are used for men and women to define if they are a binge drinker or not. The definition used here of a binge drinker is different to that of the alcohol use disorders test (AUDIT) questionnaire developed by the World Health Organisation. The AUDIT questionnaire takes gender into account asking how often an individual had six or more units if female or eight or more units if male, on a single occasion. Comparing to the AUDIT’s definition of a binge drinker, the Monitor project’s threshold for a binge drinker appears higher as measured using wine, lower for spirits and about the mid-point for beer.^1^ The quantities have been converted into centiliters of pure alcohol to allow easier comparability across alcohol types by multiplying in liter terms: beer by 4.62 %, wine by 12.8 %, and spirits by 38 % (standard measures are provided by CAN [11] and converted to % volume measures (1 cl pure alcohol is 7.8 g of alcohol). Frequency corresponds to the number of drinking episodes in the last 30 days and intensity corresponds to the amount consumed during a typical drinking episode. Quantity of alcohol consumed is the product of frequency and intensity.

The data used in our analysis covers the years 2004 through to 2011 and consists of a total of 144,025 observations. The final sample size is 126,852 after accounting for missing data (see Table 1 in Appendix 2 for details).^2^ The response rate in the period 2004–2011 fell from about 60 % to roughly between 35 and 45 % towards the end of the study period [12]. Analysis of the response rate found no systematic bias as a result of this fall in response [12]. A standard problem with surveys regarding alcohol is the lower response rate of heavy and or binge drinkers and the resulting bias in alcohol consumption estimates [13]. This survey is no exception in this regard as no compensation for this known effect was made. Summary statistics for all variables are shown in Table 1.

**Table 1:** Variable means by sample

Variable	Definition	Whole sample		Beer drinkers		Wine drinkers		Spirit drinkers	
		Male	Female	Male	Female	Male	Female	Male	Female
DRINKER	1 = Drank alcohol in last 30 days	0.82	0.73	–	–	–	–	–	–
DRINK_BEER	1 = Drank beer in last 30 days	0.60	0.25	–	–	–	–	–	–
DRINK_WINE	1 = Drank wine in last 30 days	0.54	0.62	–	–	–	–	–	–
DRINK_SPIRIT	1 = Drank spirits in last 30 days	0.53	0.26	–	–	–	–	–	–
BINGE DRINKER	1 = Binged in last 30 days	0.37	0.16	–	–	–	–	–	–
FREQUENCY	No. of days drank in last 30			5.17	3.30	5.50	5.33	3.68	2.51
INTENSITY	Average grams pure alcohol/occasion			5.51	3.25	4.27	3.97	4.52	2.95
INCOME1	1 = Monthly income less than 10,000 sek	0.11	0.19	0.09	0.15	0.05	0.13	0.08	0.17
INCOME2	1 = Monthly income 10,000–14,999 sek	0.10	0.18	0.06	0.12	0.07	0.15	0.07	0.14
INCOME3	1 = Monthly income 15,000–19,999 sek	0.14	0.20	0.12	0.21	0.12	0.20	0.13	0.20
INCOME4	1 = Monthly income 20,000–29,000 sek	0.37	0.31	0.41	0.37	0.37	0.36	0.39	0.34
INCOME5	1 = Monthly income 30,000–39,999 sek	0.16	0.08	0.19	0.10	0.21	0.10	0.19	0.10
INCOME6	1 = Monthly income 40,000 sek+	0.12	0.04	0.13	0.05	0.18	0.05	0.14	0.05
COMPULSORY SCHOOL	1 = Left school after compulsory education (aged 16 years)	0.28	0.26	0.22	0.17	0.19	0.20	0.24	0.21
COLLEGE	1 = Finished college education (aged 19 years)	0.40	0.35	0.45	0.40	0.38	0.35	0.42	0.36
UNIVERSITY	1 = Finished University education	0.32	0.38	0.33	0.43	0.42	0.45	0.34	0.43
AGE	Age in years	47.85	49.40	44.69	44.70	49.30	49.26	47.22	46.81
COHABIT	1 = Cohabits with one or more adults	0.74	0.69	0.75	0.73	0.78	0.71	0.75	0.71
EMP	1 = Currently employed	0.68	0.63	0.77	0.75	0.74	0.69	0.73	0.68
UNEMP	1 = Currently unemployed	0.03	0.03	0.02	0.03	0.02	0.02	0.02	0.03
STUDENT	1 = Currently studying full-time	0.08	0.08	0.07	0.10	0.04	0.07	0.06	0.11
INACTIVE	1 = Currently inactive (not seeking work, retired)	0.22	0.26	0.13	0.12	0.20	0.22	0.19	0.19
*N*		59,251	67,601	35,649	17,144	31,927	42,217	31,243	17,731

Monitor project data, 2004–2011. 10,000 sek in 2014 was roughly equivalent to $1600 or €1150

#### Aggregate national price indices and changes in alcohol excise rates

**Fig. 1:**
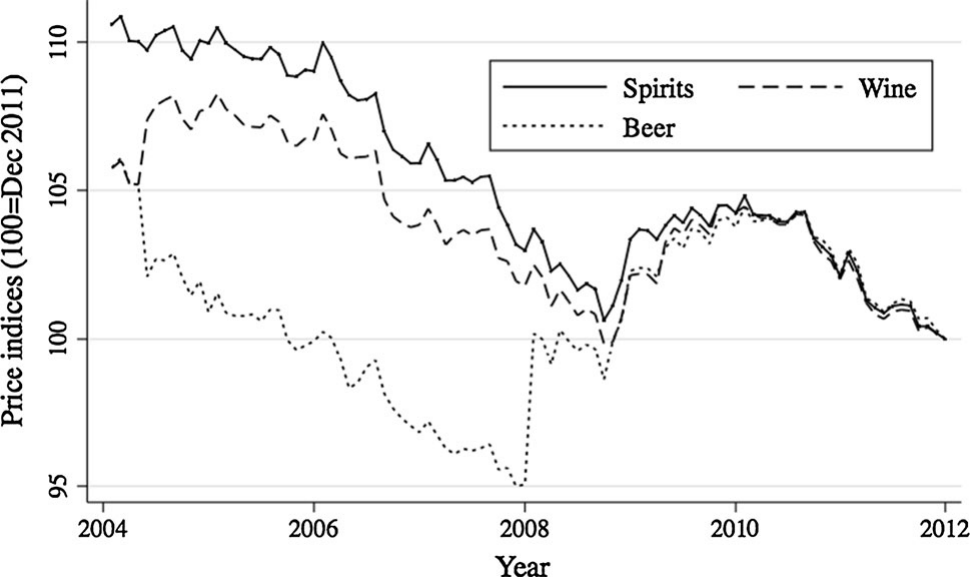
Monthly real alcohol price indices 2004–2011. *Notes* Data source: SCB, consumer price index subcategory indices for beer, wine and spirits (as per COICOP definition) are deflated by the headline consumer price index and each is rebased to December 2011 prices so that each index = 100 in December 2011

National alcohol price indices for wine, spirits, and beer (shown in Fig. 1) are provided by Statistics Sweden (Statistika Centralbyrån). These price indices are monthly and have been deflated by the CPI index (from Statistics Sweden) so that each index is in December 2011 prices and rebased so that they all equal 100 in December 2011. There is no overall price trend for beer but wine and spirits have seen a fall in real prices over the 7-year period. On January 1, 2008, alcohol duty was raised by about 13 % for beer and about −2 % for wine, and remained unchanged for spirits [14]. We use this exogenous variation in the analysis by means of an interrupted time-series dummy (“Alcohol duty change 08”) for the duty change on January 1, 2008. There is a strong correlation between wine and spirit prices (correlation coefficient of 0.96) and a weak correlation between beer and wine (0.06) and beer and spirits (0.15). To give an idea of the relative prices for each alcohol type, the excise duty rates in equivalent 100 % volume/liter terms in 2015 were 194 sek for beer, 196 sek for wine (assuming a bottle of wine is 12.8 %) and 511 sek for spirits [14]. While this is not the full picture of the cost of alcohol, it does clearly show that spirits are an expensive way to consume alcohol in Sweden.

### Methods

#### Frequency and intensity

The aim of the analysis is to estimate the determinants of demand for frequency and intensity. We start by assuming that frequency and intensity have differential impacts on individual utility. Let frequency, *F*, be defined as the total number of days in which an individual drank in the last 30 days and intensity, *I*, be defined as the average quantity drunk across all drinking sessions in the last 30 days of type *k* alcohol consumed. In addition, let *X* be a matrix of covariates observed alongside *F* and *I,* where *X* includes a constant (column of 1 s), a linear time trend (month), an interrupted time-series dummy (“Alcohol duty change 08″) that equals one after the duty change on 1st of January 2008 (own prices (*P*_*k*_) are included in separate regression equations which are provided in addition to the main results and these exclude the alcohol duty change dummy),^3^ net monthly income (*Y*) and individual characteristics, *Z*, that also affect the alcohol consumption decision together yields the following two demand equations for frequency and intensity (for ease of notation we omit the subscripts for the *k* types of alcohol):

1 $$\ln I = \ln X^{{\prime }} \beta_{I} + v_{I}$$

2 $$\ln F = \ln X^{\prime } \beta_{F} + v_{F}$$

where ln *F* and ln *I* are observed if and only if the individual chooses to drink (the participation equation is set out below). The advantage of the log–log demand equation is that interpretation is relatively straightforward: the coefficient corresponding to price in the vector *β* for example is a price elasticity: a 1 % change in price leads to a *β* % change in frequency/intensity consumed.^4^

The multiplication of frequency and intensity as defined here equals the total quantity consumed in the last 30 days, (*Q* = *F* · *I*) and is the definition used for quantity in our data. Letting *Q* be the quantity of type *k* alcohol consumed, yields the log–log demand equation for quantity:

3 $$\ln Q = \ln X^{{\prime }} \beta_{Q} + v_{Q}$$

Substituting ln *Q* with the expressions for ln *I* + ln *F* into (3) yields:

4 $$\ln Q = \ln I + \ln F = \ln X^{'} \left( {\beta_{I} + \beta_{F} } \right) + v_{I} + v_{F}$$

In our empirical analysis, we assume a log–log relationship between frequency/intensity and the explanatory variables. Equation (4) shows that the coefficients for the natural log of quantity equation will equal the sum of the coefficients for the natural log of the sum of frequency and intensity. As long as the assumption of a log–log relationship between the covariates and quantity, frequency, and intensity holds, then we can expect that on average we can interpret the results for quantity as per Eq. (4). In our empirical analysis, we estimate Eqs. (1), (2), and (3).

#### Empirical approach

A correction for sample selection is included in the demand equations. This is because a large number of individuals with zero alcohol consumption or zero episodes of binge drinking are observed and this is the result of an explicit decision not to drink or binge drink. For some individuals, the decision not to drink is absolute, and under no circumstances would they change their mind, for religious reasons for example. For others, however, there may be circumstances where they would participate; if prices fell to a low enough level or their disposable income increased, for example. For this group, it is possible that the error terms of the participation and the quantity demanded equations are correlated and standard OLS of frequency/intensity demanded would be inconsistent in this case. A type II Tobit is used to control for sample selection endogeneity that relies on the functional form assumed for the error term of the selection equation. This is a strong identifying assumption, but it does mean we do not have to identify an exclusion restriction, itself a difficult empirical issue because it is not clear which factors are associated with participation and not the frequency/intensity decisions. The Heckman two-step method [16] assumes that the error term from the selection equation *ɛ*_1_ is standard normal and therefore participation, *D*, is estimated via a Probit. This then yields the conditional mean for *Q*, given participation (similarly for frequency and intensity):

5 $$\begin{aligned} {\text{E}}\left[ {Q |X,D^{*} > 0} \right] &=X^{'} \beta_{2} + \delta {\text{E}}\left[ {\mu |\mu > - X^{'} \beta_{1} } \right] \\ &= X^{'} \beta_{2} + \delta \lambda \left( {X^{'} \beta_{1} } \right) \\ \end{aligned}$$

where *D* ^*^ is an unobserved latent variable representing a drink participation preference parameter that is greater than zero when individuals are observed drinkers (*D* = *1*) [17], *δ* is the covariance of the selection equation error term *μ* and the quantity equation error term *v*_*Q*_. *λ*(·) is the inverse Mills Ratio (IMR) or the hazard ratio where $$\lambda = {\raise0.7ex\hbox{${\phi ( \cdot )}$} \!\mathord{\left/ {\vphantom {{\phi ( \cdot )} {\varPhi ( \cdot )}}}\right.\kern-0pt} \!\lower0.7ex\hbox{${\varPhi ( \cdot )}$}}$$ and represents the probability of being censored assuming *ɛ*_1_ is distributed standard normal. The key assumption is:

6 $$v_{Q} = \delta \mu + \xi ,$$

where *ξ* is an error term and E(*ξ*|*μ*) = 0. Thus unobserved heterogeneity in the quantity (frequency and intensity) equation is accounted for through the correlation between the error terms. If *δ* is zero, then the model becomes just a double hurdle model. Information is provided on the range of probit predictions to help assess how well the functional form assumption is predicting the extreme probabilities in order to give an indication of how likely the IMR is to be identified in the quantity, frequency, and intensity equations.

The form of the quantity/frequency/intensity equations has been outlined above (Eqs. 1–3) and are estimated using Eq. (5) providing the impact of the covariates conditional on a positive outcome. In our analysis, we estimate only the conditional effects of the covariates on frequency and intensity because combining the participation effects with the frequency and intensity effects to estimate the unconditional marginal effects would hide important differences between the frequency and intensity responses, and it is these differences that are of interest. For the binary choice of participation/non-participation, we consider two overlapping groups of drinkers; the population of all drinkers (which as a group include binge drinkers) and the sub-group of binge drinkers. The participation equation for all drinkers, where *D*_All_ = 1 for a drinker who has alcohol consumption greater than zero in the last 30 days, is given by:

7 $$D_{\text{All}} = X^{{\prime }} \beta_{\text{All}} + u_{\text{All}} ,$$

where the matrix *X* contains the same explanatory variables as for the frequency and intensity equations.

For binge drinkers, the participation equation is the same as Eq. (7) but now participation is defined as *D*_Binge_ = 1 for those who in the last 30 days had at least one binge drinking episode (see the data description for the exact definition), 0 if not a binge drinker (not a binge drinker includes non-drinkers and drinkers who do not binge drink). In this case, we are controlling for sample selection into binge drinking in order to assess the frequency and intensity decisions of this sub-group. There are potentially many reasons for unobservables in the binge participation equation to be correlated with the frequency and intensity of binge drinkers. These could include a preference for getting drunk or a lack of control once one has started drinking. However, due to the limitations of the data, we do not have information on such preferences and therefore we rely on the same functional form assumption of the Heckman two-step procedure to identify the inverse Mills ratio, as we have done for all drinkers, in order to control for the potential unobserved correlation in the error terms.

#### Exogenous variation

In general, prices are assumed to be endogenous in a demand system equation. This is because while prices affect demand, they are also affected by supply. In Sweden, alcohol is highly regulated, and the only off-license is the national off-license monopoly (Systembolaget). In 2012, 63 % of alcohol sales were through Systembolaget [12] and Sweden has excise duties on alcohol amongst the highest in the world. The normal demand and supply relationship is therefore highly distorted by government taxation and regulation. It is therefore argued that the price index used can be seen as exogenously determined. Excise duties for beer in fact saw a large jump in January 2008 (up 13 %) and wine saw a small drop (−2 %). In our main analysis, we exploit this by estimating an interrupted time-series model including a linear time trend, leaving analysis of prices to the Appendix (due to the high colinearity of the price indices and remaining potential endogeneity issues we include the results for prices purely for reference).

Potentially, more troublesome are the variables income, employment status, and to a lesser extent, education. The empirical issues are summarized in Cook and Moore [18]. A common result in the literature is that those who earn more are more likely to drink, and drink more than those who earn less. This has been thought to be a problem of misclassification of non-drinkers, as many non-drinkers are previous heavy drinkers, although Jarl and Gerdtham [19] still found the same relation after controlling for this. For most individuals in the sample, their education level will have been previously established and therefore simultaneity is unlikely to be a big problem. However, there may be a third variable that affects both the education decision and the alcohol preference—possibly a risk preference that we are unable to control for. For these variables, it is only possible to describe the observed association.

## Results

The results are estimated for males and females separately due to the very different patterns of alcohol consumption observed between the genders. The regression results are presented for males, and where differences are observed between the genders, these are discussed (results for females are found in Appendix 1). In general, the regression results show that between the years 2004 and 2011 there has been a downward trend in drinking participation, participation in binge drinking, frequency, and intensity of drinking episodes as estimated by a linear trend function (controlling for covariates). More men than women drank alcohol, and on average men and women had similar wine drinking patterns, but men drank more beer and spirits and were more likely to binge drink (see Table 1).

The results of the alcohol participation decision are presented in Appendix 1 (Table 5 for males and Table 7 for females) and are presented as average partial effects. The results show that income and education were positively correlated with the binge drinking decision across all types of alcohol, but less so in comparison to all drinkers. The highest income group was 27 % more likely to participate in beer consumption in comparison to the lowest income group. This likelihood is slightly higher for wine participation but about half as large for binge drinkers. A much greater distinction between binge drinkers and all drinkers is observed for the covariates of both age and economic activity where being younger and economically inactive are both stronger predictors for binge drinking compared to all drinkers. All covariates are fairly similar in magnitude across the alcohol types for binge drinkers, suggesting binge drinkers distinguished to a lesser extent between the alcohol types during the decision process to binge drink. Women and men had statistically different values for the explanatory variables in the wine participation equation, but these were fairly similar in size in a broader economic sense. Women had much shallower income and education gradients for beer and spirits consumption participation. Turning to price, the excise duty rate increase in January 2008, as assessed by interrupted time-series, had a negative impact on participation across the alcohol types (varying between −1 and −2 % depending on alcohol type and drinking group), with the exception of wine, which consistently saw no significant impact by gender and drinking group. The additional regressions for participation that include price, but not the excise duty rate dummy variable (see Tables 6 and 8 in Appendix 1), find generally positive but largely insignificant own price elasticities for all alcohol types and drinker types and the effect sizes are very large, ranging between −9 and 37 % for a 1 % change in price levels (controlling for a linear trend).

The results for the demand for frequency and intensity *conditional* on participation are shown in Tables 2–4 (Tables 10–12 in Appendix 1 for females). The fitted values of the inverse Mills ratio (IMR) have a positive and statistically significant effect on the beer and wine frequency decision but for men only. Sample selection appears to be a bigger concern for binge drinkers with much larger IMR values (again only for males). The IMR values for binge drinkers suggest that those that select to be binge drinkers have a higher frequency of beer and spirits consumption, lower beer intensity consumption, and a higher spirits intensity consumption compared to a random draw from the population. As shown in the participation equations [Appendix, Tables 5, 6 (males) and 7, 8 (females)], there are mixed successes in the Probit model’s ability to predict extreme low and high probabilities, especially for women. Therefore, where the IMR is not significant (in Tables 2–4), this does not necessarily suggest that unobserved heterogeneity is not an issue. It is possible that there remains unobserved heterogeneity that is correlated with the errors due to sample selection for the equations where the IMR is non-significant.

**Table 2:** Beer frequency and intensity demand equation estimates, males

	All drinkers		Binge drinkers	
	Frequency	Intensity	Frequency	Intensity
Linear time trend	−0.01	−0.00	−0.02*	0.01
	(0.00)	(0.00)	(0.01)	(0.01)
Alcohol duty change 08	−0.05**	0.04***	−0.07**	0.07***
	(0.02)	(0.01)	(0.03)	(0.02)
INCOME1 (reference)				
INCOME2	0.07	0.00	0.12*	−0.06
	(0.04)	(0.03)	(0.06)	(0.05)
INCOME3	0.23***	−0.03	0.28***	−0.14**
	(0.07)	(0.06)	(0.09)	(0.07)
INCOME4	0.37***	−0.02	0.46***	−0.23**
	(0.11)	(0.08)	(0.14)	(0.10)
INCOME5	0.47***	−0.08	0.56***	−0.36***
	(0.12)	(0.09)	(0.16)	(0.12)
INCOME6	0.48***	−0.16*	0.57***	−0.47***
	(0.13)	(0.09)	(0.15)	(0.12)
COMPULSORY SCHOOL (reference)				
COLLEGE	0.07***	−0.01	0.15***	−0.11***
	(0.03)	(0.02)	(0.05)	(0.04)
UNIVERSITY	−0.02	−0.14***	0.01	−0.18***
	(0.02)	(0.01)	(0.02)	(0.02)
LNAGE	−0.15*	−0.83***	−0.54**	−0.25
	(0.08)	(0.06)	(0.24)	(0.18)
EMP (reference)				
INACTIVE	−0.12**	−0.17***	−0.29**	0.04
	(0.05)	(0.04)	(0.12)	(0.09)
UNEMP	0.05	0.08***	0.03	0.09**
	(0.03)	(0.02)	(0.05)	(0.04)
STUDENT	−0.02	−0.10***	−0.07	−0.03
	(0.03)	(0.02)	(0.05)	(0.04)
COHABIT	−0.02	−0.20***	−0.11***	−0.11***
	(0.01)	(0.01)	(0.03)	(0.03)
IMR	0.58**	−0.18	0.97***	−0.71***
	(0.25)	(0.19)	(0.34)	(0.26)
CONSTANT	1.25***	4.86***	2.24***	3.59***
	(0.08)	(0.06)	(0.48)	(0.36)
Participation observations	59,251	59,251	59,251	59,251
Freq/intens observations	35,650	35,650	19,297	19,297
Proportion drink/binge	60 %	60 %	33 %	33 %

Results are conditional on beer drinking participation. Reference categories are income <10,000 sek, compulsory schooling, employed, and living alone. Alcohol duty change is a dummy set to one after January 1, 2008, when there was a change in beer and wine excise duties. Month dummies to capture resampling effects and seasonality and regional dummies are included in all models (reference categories: Stockholm and January). Testing the null of no effect: *** p < 0.01; ** p < 0.05; * p < 0.1

**Table 3:** Wine frequency and intensity demand equation estimates, males

	All drinkers		Binge drinkers	
	Frequency	Intensity	Frequency	Intensity
Linear time trend	−0.02***	−0.01***	0.04**	−0.01
	(0.01)	(0.00)	(0.02)	(0.01)
Alcohol duty change 08	−0.01	0.02*	0.03	0.01
	(0.02)	(0.01)	(0.06)	(0.02)
INCOME1 (reference)				
INCOME2	0.05	0.00	−0.27**	−0.03
	(0.05)	(0.03)	(0.14)	(0.07)
INCOME3	0.29***	0.03	−0.52**	−0.02
	(0.06)	(0.03)	(0.23)	(0.12)
INCOME4	0.52***	0.04	−0.83**	−0.01
	(0.08)	(0.05)	(0.39)	(0.20)
INCOME5	0.82***	0.05	−0.96*	0.01
	(0.11)	(0.06)	(0.51)	(0.26)
INCOME6	1.06***	0.05	−0.81	0.02
	(0.12)	(0.07)	(0.52)	(0.27)
COMPULSORY SCHOOL (reference)				
COLLEGE	0.65***	−0.00	−0.14	0.08
	(0.06)	(0.04)	(0.21)	(0.11)
UNIVERSITY	0.96***	−0.04	1.37***	0.11
	(0.05)	(0.03)	(0.28)	(0.14)
LNAGE	0.14***	−0.14***	0.74***	−0.12
	(0.02)	(0.01)	(0.26)	(0.14)
EMP (reference)				
INACTIVE	0.08*	0.08***	−0.10	0.08**
	(0.04)	(0.02)	(0.09)	(0.04)
UNEMP	0.42***	−0.04	−0.06	−0.03
	(0.06)	(0.03)	(0.11)	(0.05)
STUDENT	0.28***	−0.07***	0.18***	−0.08***
	(0.03)	(0.02)	(0.03)	(0.01)
COHABIT	−0.11***	0.03	0.30*	0.01
	(0.03)	(0.02)	(0.16)	(0.08)
IMR	1.04***	−0.07	−2.08**	0.14
	(0.17)	(0.10)	(0.92)	(0.48)
CONSTANT	−3.98***	1.58***	−0.95**	0.94***
	(0.41)	(0.24)	(0.42)	(0.21)
Participation observations	59,251	59,251	59,251	59,251
Freq/intens observations	31,925	31,925	14,414	14,414
Proportion drink/binge	54 %	54 %	24 %	24 %

Results are conditional on wine drinking participation. Reference categories are income <10,000 sek, compulsory schooling, employed, and living alone. Alcohol duty change is a dummy set to one after January 1st 2008 when there was a change in beer and wine excise duties. Month dummies to capture resampling effects and seasonality and regional dummies are included in all models (reference categories: Stockholm and January). Testing the null of no effect: *** p < 0.01; ** p < 0.05; * p < 0.1

**Table 4:** Spirits frequency and intensity demand equation estimates, males

	All drinkers		Binge drinkers	
	Frequency	Intensity	Frequency	Intensity
Linear time trend	−0.01	−0.02**	−0.07***	−0.07***
	(0.01)	(0.01)	(0.03)	(0.02)
Alcohol duty change 08	−0.06**	0.01	−0.25***	−0.12
	(0.02)	(0.02)	(0.09)	(0.08)
INCOME1 (reference)				
INCOME2	−0.06	−0.00	0.29*	0.27**
	(0.06)	(0.05)	(0.16)	(0.13)
INCOME3	−0.00	0.07	0.64**	0.47**
	(0.12)	(0.10)	(0.25)	(0.21)
INCOME4	0.06	0.12	1.08***	0.77**
	(0.17)	(0.15)	(0.39)	(0.32)
INCOME5	0.12	0.12	1.45***	0.96**
	(0.22)	(0.19)	(0.49)	(0.40)
INCOME6	0.18	0.09	1.55***	0.92**
	(0.24)	(0.20)	(0.50)	(0.41)
COMPULSORY SCHOOL (reference)				
COLLEGE	−0.00	−0.28***	0.05	−0.33***
	(0.03)	(0.03)	(0.06)	(0.05)
UNIVERSITY	0.26***	−0.43***	−1.53**	−1.75***
	(0.04)	(0.03)	(0.64)	(0.53)
LNAGE	0.18***	0.01	−0.41	−0.42**
	(0.02)	(0.02)	(0.25)	(0.21)
EMP (reference)				
INACTIVE	0.10**	0.13***	0.11	0.11
	(0.04)	(0.03)	(0.12)	(0.10)
UNEMP	0.11**	−0.04	−0.04	−0.22**
	(0.06)	(0.05)	(0.12)	(0.10)
STUDENT	−0.05*	−0.12***	−0.26***	−0.31***
	(0.02)	(0.02)	(0.09)	(0.07)
COHABIT	−0.05	−0.07	−0.65***	−0.44**
	(0.09)	(0.08)	(0.21)	(0.17)
IMR	−0.05	0.38	3.14***	2.57***
	(0.50)	(0.43)	(1.09)	−0.89
CONSTANT	0.07	2.76***	2.93***	5.12***
	(0.35)	(0.30)	(1.06)	(0.87)
Participation observations	59,251	59,251	59,251	59,251
Freq/intens observations	31,243	31,243	16,641	16,641
Proportion drink/binge	53 %	53 %	28 %	28 %

Results are conditional on spirit drinking participation. Reference categories are income <10,000 sek, compulsory schooling, employed, and living alone. Alcohol duty change is a dummy set to one after January 1, 2008, when there was a change in beer and wine excise duties. Month dummies to capture resampling effects and seasonality and regional dummies are included in all models (reference categories: Stockholm and January). Testing the null of no effect: *** p < 0.01; ** p < 0.05; * p < 0.1

The impact of the alcohol duty rate changes in 2008 reduced the frequency of beer consumption by 5 % but raised the intensity of drinking episodes by 4 %. An impact is also observed for beer binge drinkers where frequency reduced by 7 % but intensity increased by 7 %. It appears that the excise duty change for beer also either had an income effect and subsequent substitution effect or complementary effect as it negatively impacted on the demand for frequency and intensity of spirits. The impact of own prices on frequency and intensity appears to be negative for frequency (see Table 9 in Appendix 1). Significant and large impacts of own prices are observed for frequency of binge drinkers of wine and beer for males but not for females. Similarly to the participation equations, the impacts of own prices are very large in comparison to the impacts of the alcohol duty changes and other covariates.

Broadly, across the alcohol types there was a positive income gradient to frequency demand and a negative income gradient to intensity demand. The highest income group had a 48 % higher frequency of beer consumption and 16 % lower intensity of beer consumption in comparison to the lowest income group. Binge beer drinkers had a steeper income gradient in both frequency and intensity. The income pattern for spirits drinkers was similar to that of beer consumption with regards to frequency. Unlike beer, spirits had a positive income gradient in intensity. Higher education levels predict lower levels of intensity for beer and spirits consumption, but higher levels of frequency of beer and wine consumption. Women differ slightly from this pattern in that there is no clear relationship between income, education and frequency or intensity for drinkers of beer and spirits.

Most often, where a positive gradient for income and education is observed for the quantity equation (see Appendix, Tables 14, 15, 16, 17), this is driven by the positive income and education gradients in frequency demand dominating the negative income and education gradients in intensity demand and vice versa. Where no statistically significant relation is observed for the quantity equation, this can obscure important responses. For example, the income response of beer binge drinkers shows a positive income gradient for frequency and negative gradient for intensity but no significant effect for quantity.

## Discussion

This paper has broadened the evidence base regarding the determinants of frequency and intensity demand for alcohol using a large individual level dataset from Sweden that has allowed the analysis to be extended to different alcohol types, drinker types, and to be split by gender. The time period under analysis is interesting, as it is a period where a reduction in drinkers, binge drinkers, quantity, frequency, and intensity of alcohol consumption has been observed. These trends are important to note as they involved large reductions in consumption generally during a period of relatively small changes in the real prices of alcohol. Real (inflation-adjusted) prices of beer, wine, and spirits, in general, fell at the same time that the proportion of the population who drank and the intensity in which they consumed fell. With the entry to the EU, Sweden saw increasing liberalization of alcohol trade with other EU members and alcohol private import quotas were fully abolished by 2004. Alcohol consumption had been increasing up until 2004, but since 2004 overall alcohol consumption has been on the decline [12]. Our results generally find large positive but largely insignificant own price elasticities. The participation price elasticities are significant for binge drinkers of wine and spirits, but this cannot be a realistic result. More likely is that preferences for alcohol by binge drinkers has changed in Sweden in a way that cannot adequately be explained by changes in price, controlling for other observables (including a linear time trend to capture the general decline in consumption patterns), and that we have not been able to explain these changing preferences adequately.

We now turn to the impact of the change in excise duty and its analysis using interrupted time-series analysis. It appears that the alcohol excise duty increase enacted in January of 2008 did reduce beer drinking participation and frequency of consumption but raised the intensity. It also appears to have reduced both intensity and frequency of wine and spirits consumption. The results of the excise duty rate change on beer consumption are similar to those of Byrnes et al. [9] who found a negative price elasticity of frequency of light drinking days. However, we find that the impact on binge drinkers was similar to that for all drinkers, but stronger for males, and Byrnes et al. [9] found no impact on frequency of heavy drinking days. Why there was a positive intensity response to the excise rate change is not clear. The price response to the excise duty change observed here suggests that individuals respond to increased prices by drinking less often but substitute somewhat by increasing how intensely they drink. We also observe that the excise rate change for beer had no measurable effect on quantity consumed, yet it increased the intensity of consumption, highlighting the importance of frequency and intensity analysis. It is important to note that interrupted time-series analysis attempts to capture the effect of a time-specific policy, but due to a lack of a control group, it is possible that other events occurred at the same time as the change in excise rates that we are unable to control for.

Previous analysis of the determinants of frequency and intensity of alcohol consumption [2] concluded that income and education are negatively associated with intensity and that neither had an effect on frequency. The results of the current paper find that generally across different alcohol types and different types of drinkers there was a positive income and education gradient with frequency and a negative income and education gradient with intensity. Broadly, we find income and education had a positive gradient with drinking and binge drinking participation, quantity and frequency demanded, but a negative gradient with intensity demanded. The results presented here suggest the reason behind the positive income gradient with alcohol demand documented in Cook and Moore [18] is due to a frequency relation. A potential explanation for the observed positive relationship between income and drinking frequency could be that higher-earning individuals can afford to socialize more often and socializing norms involve the consumption of alcohol.

The key difference between men’s and women’s drinking patterns is that women predominantly drank wine. Relatively few women drank beer or spirits (in grams of alcohol terms) whereas men were more evenly split amongst the alcohol types. Wine is very different to beer and spirits in terms of who drinks it and who drinks it more frequently and intensely. Income and education were positively associated with both frequency and intensity demand for wine. Wine appears to be a luxury good, favored by higher earners and the more educated, especially women.

The results for the sub-group of binge drinkers show important differences to the population of drinkers as a whole. Participation in binge drinking is strongly predicted by young age and economic inactivity. Binge drinkers appeared to differentiate between the alcohol types to a lesser degree in comparison to average drinkers, possibly reflecting a different attitude to alcohol. Across all alcohol types the more educated bingers drank less intensely, possibly reflecting the increased opportunity cost of intense drinking episodes and/or greater health awareness, but they drank more often.

As set out in the introduction, binge drinkers are associated with higher social costs. The results presented here highlight the complexities associated with attempting to limit the social costs of this harmful drinking behavior. An alcohol-related policy targeted at the less educated would, for example, have to understand why the less educated are less likely to binge drink but the intensity of less educated individuals who binge drink is higher. Policy aimed at reducing socioeconomic-related health inequalities needs to consider the particular complexities of alcohol demand highlighted in this study. Previous research investigating various socioeconomic measures related to alcohol participation inequality in Sweden by Combes et al. [20] has found alcohol participation to be positively associated with income. This is consistent with the findings of the current paper. However, the demand for intensity by binge drinkers is negatively associated with education and income. Given that binge drinkers who drink most intensely are the individuals who will have the worse wider health outcomes, the findings of this paper suggest it is in fact individuals with low education and low income levels who drink more intensely that should be the focus of policy, contrary to Combes et al [20]. In fact, a newly designed randomized trial in the UK [21] is focusing exactly on this group, highlighting how the results presented here could help focus policy interventions.

The results presented in this paper are robust associations and are useful for highlighting which groups of individuals drink in the most harmful way and where alcohol policy could most effectively be targeted in order to reduce alcohol-related harm and its negative impact on health. However, the results are not free from endogeneity or simultaneity. It therefore cannot be said that policy aimed at changing the factors observed in this study, such as education levels, will change alcohol drinking behavior as might be expected if the results were interpreted as causal effects. Addressing the potential endogeneity and two-way causality issues that potentially exist in the analysis presented here is a valuable line of investigation for future research.

The results of this paper are subject to a few further limitations that impact the potential interpretation of the results. While the data used in this paper are very detailed and have enabled us to examine in more detail the demand for frequency and intensity, the main limitation of the paper is the quality of the data we have used. This reflects the challenging nature of alcohol demand analysis. As noted in the data description, the Monitor project data saw a reduction in response rate with a response rate near the end of the survey period in the range of 35–45 %. There is also evidence of missing values bias where those who report an income are more educated, male, younger, and more likely to be employed and those who fail to report their drinking behaviors are younger, with lower income and who are male. The Monitor project also made no effort to counter the fact that heavy drinkers are less likely to respond to survey questionnaires. There has been research looking into the response rate of the survey data and this found no significant issues, but nonetheless, a response rate of 35 % will always be a concern, and the results found in this paper could just reflect a subset of the population who are willing to respond to such a survey. It is also concerning that missing values can be predicted by covariates, and beyond controlling for these covariates the results have to be interpreted with caution, as there may be other factors that both predict alcohol drinking behavior and non-response to a particular question. Finally, our definition of binge drinker is not the same as the more internationally recognized definition used in the AUDIT survey. This may potentially explain differences between the results found here and those found elsewhere, especially as the definition used in this paper does not distinguish between genders and the differences we observe between genders could be driven by our definition.

## Appendix 1: Supplementary results

See Tables 5, 6, 7, 8, 9, 10, 11, 12, 13, 14, 15, 16 and 17.

**Table 5:** Drinking participation equations, males

Variables	Alcohol selection equation			Binge drinking selection equation		
	Beer	Wine	Spirits	Beer	Wine	Spirits
Linear time trend	−0.00***	−0.01***	−0.01***	−0.01***	−0.01***	−0.01***
	(0.00)	(0.00)	(0.00)	(0.00)	(0.00)	(0.00)
Alcohol duty change 08	−0.01*	0.00	−0.02***	−0.02***	−0.01*	−0.02***
	(0.01)	(0.01)	(0.01)	(0.01)	(0.01)	(0.01)
INCOME1 (reference)						
INCOME2	0.08***	0.07***	0.06***	0.05***	0.03***	0.04***
	(0.01)	(0.01)	(0.01)	(0.01)	(0.01)	(0.01)
INCOME3	0.16***	0.13***	0.13***	0.08***	0.06***	0.07***
	(0.01)	(0.01)	(0.01)	(0.01)	(0.01)	(0.01)
INCOME4	0.23***	0.21***	0.19***	0.14***	0.12***	0.12***
	(0.01)	(0.01)	(0.01)	(0.01)	(0.01)	(0.01)
INCOME5	0.27***	0.29***	0.24***	0.15***	0.15***	0.14***
	(0.01)	(0.01)	(0.01)	(0.01)	(0.01)	(0.01)
INCOME6	0.27***	0.33***	0.25***	0.15***	0.15***	0.14***
	(0.01)	(0.01)	(0.01)	(0.01)	(0.01)	(0.01)
COMPULSORY SCHOOL (reference)						
COLLEGE	0.06***	0.12***	0.04***	0.05***	0.07***	0.04***
	(0.00)	(0.00)	(0.00)	(0.00)	(0.00)	(0.00)
UNIVERSITY	0.01**	0.21***	0.04***	0.00	0.08***	0.01
	(0.01)	(0.00)	(0.01)	(0.00)	(0.00)	(0.00)
LNAGE	−0.20***	0.17***	−0.04***	−0.31***	−0.11***	−0.24***
	(0.01)	(0.01)	(0.01)	(0.01)	(0.01)	(0.01)
EMP (reference)						
INACTIVE	−0.09***	0.01	0.02***	−0.12***	−0.09***	−0.07***
	(0.01)	(0.01)	(0.01)	(0.01)	(0.01)	(0.01)
UNEMP	−0.00	0.02*	0.02*	0.01	0.01	0.00
	(0.01)	(0.01)	(0.01)	(0.01)	(0.01)	(0.01)
STUDENT	−0.02**	0.14***	0.05***	−0.04***	0.03**	−0.02**
	(0.01)	(0.01)	(0.01)	(0.01)	(0.01)	(0.01)
COHABIT	0.02***	0.09***	0.03***	−0.04***	−0.01**	−0.03***
	(0.00)	(0.00)	(0.00)	(0.00)	(0.00)	(0.00)
Predicted min	0.09	0.05	0.17	0.01	0.01	0.01
Predicted max	0.93	0.95	0.87	0.89	0.69	0.82
*N*	59,251	59,251	59,251	59,251	59,251	59,251

Estimates are average partial effects. Dummies from same category are estimated with all other dummies in same category set to zero. Reference categories are income <10,000 sek, compulsory schooling, employed, and living alone. Alcohol duty change is a dummy set to one after January 1, 2008, when there was a change in beer and wine excise duties. Month dummies to capture resampling effects and seasonality and regional dummies are included in all models (reference categories: Stockholm and January). Testing the null of no effect: *** p < 0.01; ** p < 0.05; * p < 0.1

**Table 6:** Drinking participation equations, males (price indices results)

Variables	Alcohol selection equation			Binge drinking selection equation		
	Beer	Wine	Spirits	Beer	Wine	Spirits
Linear time trend	−0.01***	−0.01***	−0.02***	−0.01***	−0.01***	−0.01***
	(0.00)	(0.00)	(0.00)	(0.00)	(0.00)	(0.00)
Ln (beer price index)	0.01			0.02		
	(0.07)			(0.06)		
Ln (wine price index)		0.19			0.27**	
		(0.13)			(0.11)	
Ln (spirit price index)			0.15			0.31**
			(0.15)			(0.12)
*N*	59,251	59,251	59,251	59,251	59,251	59,251

See notes for Table 5. Results for income, education, employment status, and cohabiting are not shown but are included in the regression. The excise duty rate change in January 2008 is not controlled for in these results

**Table 7:** Drinking participation equations, females

Variables	Alcohol selection equation			Binge drinking selection equation		
	Beer	Wine	Spirits	Beer	Wine	Spirits
Linear time trend	−0.01***	−0.01***	−0.01***	−0.00***	−0.00***	−0.01***
	(0.00)	(0.00)	(0.00)	(0.00)	(0.00)	(0.00)
Alcohol duty change 08	−0.01**	0.00	−0.01	−0.01**	−0.00	−0.01*
	(0.01)	(0.01)	(0.01)	(0.00)	(0.00)	(0.00)
INCOME1 (reference)						
INCOME2	0.02***	0.08***	0.02***	0.02***	0.02***	0.02***
	(0.01)	(0.01)	(0.01)	(0.00)	(0.00)	(0.00)
INCOME3	0.06***	0.13***	0.05***	0.02***	0.03***	0.03***
	(0.01)	(0.01)	(0.01)	(0.00)	(0.00)	(0.00)
INCOME4	0.09***	0.20***	0.08***	0.04***	0.06***	0.04***
	(0.01)	(0.01)	(0.01)	(0.00)	(0.01)	(0.00)
INCOME5	0.10***	0.26***	0.12***	0.05***	0.07***	0.06***
	(0.01)	(0.01)	(0.01)	(0.01)	(0.01)	(0.01)
INCOME6	0.12***	0.28***	0.12***	0.05***	0.08***	0.06***
	(0.01)	(0.01)	(0.01)	(0.01)	(0.01)	(0.01)
COMPULSORY SCHOOL (reference)						
COLLEGE	0.05***	0.10***	0.02***	0.03***	0.06***	0.03***
	(0.00)	(0.00)	(0.00)	(0.00)	(0.00)	(0.00)
UNIVERSITY	0.03***	0.15***	0.03***	0.01***	0.04***	0.02***
	(0.00)	(0.00)	(0.00)	(0.00)	(0.00)	(0.00)
LNAGE	−0.07***	0.16***	−0.04***	−0.14***	−0.17***	−0.15***
	(0.01)	(0.01)	(0.01)	(0.00)	(0.00)	(0.00)
EMP (reference)						
INACTIVE	−0.11***	−0.06***	−0.01	−0.06***	−0.05***	−0.03***
	(0.01)	(0.01)	(0.01)	(0.00)	(0.00)	(0.00)
UNEMP	0.01	−0.02*	0.02*	0.01*	0.00	0.01*
	(0.01)	(0.01)	(0.01)	(0.01)	(0.01)	(0.01)
STUDENT	0.03***	0.12***	0.10***	0.02***	0.03***	0.03***
	(0.01)	(0.01)	(0.01)	(0.01)	(0.01)	(0.01)
COHABIT	0.02***	0.05***	0.02***	−0.03***	−0.04***	−0.02***
	(0.00)	(0.00)	(0.00)	(0.00)	(0.00)	(0.00)
Predicted min	0.03	0.13	0.06	0.00	0.00	0.01
Predicted max	0.52	0.94	0.61	0.47	0.55	0.49
*N*	67,601	67,601	67,601	67,601	67,601	67,601

Estimates are average partial effects. Dummies from same category are estimated with all other dummies in same category set to zero. Reference categories are income <10,000 sek, compulsory schooling, employed and living alone. Alcohol duty change is a dummy set to one after January 1, 2008, when there was a change in beer and wine excise duties. Month dummies to capture resampling effects and seasonality and regional dummies are included in all models (reference categories: Stockholm and January). Testing the null of no effect: *** p < 0.01; ** p < 0.05; * p < 0.1

**Table 8:** Drinking participation equations, females (price indices results)

Variables	Alcohol selection equation			Binge drinking selection equation		
	Beer	Wine	Spirits	Beer	Wine	Spirits
Linear time trend	−0.01***	−0.01***	−0.01***	−0.01***	−0.00**	−0.00***
	(0.00)	(0.00)	(0.00)	(0.00)	(0.00)	(0.00)
Ln (beer price index)	−0.09			0.01		
	(0.06)			(0.04)		
Ln (wine price index)		0.01			0.37***	
		(0.11)			(0.09)	
Ln (spirit price index)			0.01			0.25***
			(0.12)			(0.08)
*N*	67,601	67,601	67,601	67,601	67,601	67,601

See notes for Table 7. Results for income, education, employment status, and cohabiting are not shown but are included in the regression. The excise duty rate change in January 2008 is not controlled for in these results

**Table 9:** Frequency and intensity demand equation estimates, males (price indices results)

	All drinkers		Binge drinkers	
	Frequency	Intensity	Frequency	Intensity
Beer				
Linear time trend	−0.01***	0.00	−0.03***	0.02***
	(0.00)	(0.00)	(0.01)	(0.01)
Ln (beer price index)	−0.62***	0.10	−0.58**	0.03
	(0.19)	(0.14)	(0.28)	(0.21)
Wine				
Linear time trend	−0.03***	−0.01***	0.03	−0.01
	(0.00)	(0.00)	(0.02)	(0.01)
Ln (wine price index)	−0.54	−0.27	−2.74**	−0.40
	(0.41)	(0.21)	(1.16)	(0.47)
Spirits				
Linear time trend	−0.03**	−0.02**	−0.10***	−0.08***
	(0.01)	(0.01)	(0.03)	(0.03)
Ln (spirit price index)	−0.20	−0.16	1.96	1.19
	(0.38)	(0.34)	(1.71)	(1.35)

See notes for Table 2. For each alcohol type the results presented are from separately estimated regressions. Results for income, education, employment status, and cohabiting are not shown but are included in the regression. The excise duty rate change in January 2008 is not controlled for in these results

**Table 10:** Beer frequency and intensity demand equation estimates, females

	All drinkers		Binge drinkers	
	Frequency	Intensity	Frequency	Intensity
Linear time trend	−0.09	0.03	−0.09	0.08
	(0.05)	(0.02)	(0.06)	(0.07)
Alcohol duty change 08	−0.14	0.08	−0.17	0.25
	(0.14)	(0.06)	(0.17)	(0.19)
INCOME1 (reference)				
INCOME2	0.33	−0.07	0.51	−0.43
	(0.22)	(0.10)	(0.33)	(0.36)
INCOME3	0.75	−0.24	0.59	−0.60
	(0.51)	(0.23)	(0.42)	(0.46)
INCOME4	1.08	−0.39	1.02	−1.02
	(0.68)	(0.31)	(0.63)	(0.70)
INCOME5	1.30	−0.54	1.24	−1.40
	(0.82)	(0.37)	(0.81)	(0.89)
INCOME6	1.49	−0.63	1.39	−1.56
	(0.94)	(0.43)	(0.88)	(0.97)
COMPULSORY SCHOOL (reference)				
COLLEGE	0.53	−0.22	0.69	−0.77
	(0.34)	(0.16)	(0.43)	(0.47)
UNIVERSITY	0.35	−0.26**	0.33*	−0.45**
	(0.24)	(0.11)	(0.20)	(0.22)
LNAGE	−0.73	−0.52**	−2.66	2.13
	(0.46)	(0.21)	(1.70)	(1.87)
EMP (reference)				
INACTIVE	−1.18	0.47	−1.13	1.39
	(0.79)	(0.36)	(0.83)	(0.92)
UNEMP	0.13	0.12	0.28	−0.04
	(0.16)	(0.07)	(0.22)	(0.25)
STUDENT	0.40	−0.15	0.39	−0.48
	(0.27)	(0.12)	(0.28)	(0.31)
COHABIT	0.19	−0.25***	−0.55	0.46
	(0.12)	(0.05)	(0.37)	(0.40)
IMR	4.35	−2.00	3.67	−4.04
	(2.87)	(1.32)	(2.36)	(2.60)
CONSTANT	−2.32	5.65***	4.38**	0.46
	(2.21)	(1.02)	(2.20)	(2.42)
Participation observations	67,601	67,601	67,601	67,601
Freq/intens observations	17,148	17,148	6117	6117
Proportion drink/binge	25 %	25 %	9 %	9 %

Results are conditional on beer drinking participation. Reference categories are income <10,000 sek, compulsory schooling, employed, and living alone. Alcohol duty change is a dummy set to one after January 1, 2008, when there was a change in beer and wine excise duties. Month dummies to capture resampling effects and seasonality and regional dummies are included in all models (reference categories: Stockholm and January). Testing the null of no effect: *** p < 0.01; ** p < 0.05; * p < 0.1

**Table 11:** Wine frequency and intensity demand equation estimates, females

	All drinkers		Binge drinkers	
	Frequency	Intensity	Frequency	Intensity
Linear time trend	−0.01	−0.02***	−0.03	0.02
	(0.00)	(0.00)	(0.02)	(0.02)
Alcohol duty change 08	−0.00	0.01	−0.05	0.04
	(0.02)	(0.01)	(0.06)	(0.06)
INCOME1 (reference)				
INCOME2	0.05*	0.07***	0.26**	−0.15
	(0.03)	(0.02)	(0.13)	(0.13)
INCOME3	0.17***	0.14***	0.34*	−0.25
	(0.05)	(0.03)	(0.18)	(0.19)
INCOME4	0.30***	0.18***	0.62**	−0.45
	(0.07)	(0.04)	(0.28)	(0.28)
INCOME5	0.48***	0.19***	0.95***	−0.66*
	(0.09)	(0.05)	(0.37)	(0.37)
INCOME6	0.55***	0.19***	1.11***	−0.72*
	(0.09)	(0.06)	(0.40)	(0.41)
COMPULSORY SCHOOL (reference)				
COLLEGE	0.34***	0.02	0.63***	−0.31*
	(0.05)	(0.03)	(0.17)	(0.17)
UNIVERSITY	0.56***	−0.13***	−0.64	1.21*
	(0.06)	(0.03)	(0.67)	(0.68)
LNAGE	0.03	−0.16***	−0.39	0.40
	(0.02)	(0.02)	(0.25)	(0.26)
EMP (reference)				
INACTIVE	0.02	0.02	−0.01	0.02
	(0.03)	(0.02)	(0.09)	(0.09)
UNEMP	0.24***	0.05*	0.33**	−0.22*
	(0.05)	(0.03)	(0.13)	(0.13)
STUDENT	0.15***	−0.03**	−0.20	0.17
	(0.02)	(0.01)	(0.14)	(0.14)
COHABIT	−0.09***	−0.02	−0.46**	0.42*
	(0.03)	(0.02)	(0.22)	(0.22)
IMR	0.34	0.21	1.96*	−1.98*
	(0.21)	(0.13)	(1.05)	(1.06)
CONSTANT	−1.42***	1.59***	0.53	0.22
	(0.39)	(0.24)	(0.83)	(0.84)
Participation observations	67,601	67,601	67,601	67,601
Freq/intens observations	42,208	42,208	9114	9114
Proportion drink/binge	62 %	62 %	13 %	13 %

Results are conditional on wine drinking participation. Reference categories are income <10,000 sek, compulsory schooling, employed, and living alone. Alcohol duty change is a dummy set to one after January 1, 2008, when there was a change in beer and wine excise duties. Month dummies to capture resampling effects and seasonality and regional dummies are included in all models (reference categories: Stockholm and January). Testing the null of no effect: *** p < 0.01; ** p < 0.05; * p < 0.1

**Table 12:** Spirits frequency and intensity demand equation estimates, females

	All drinkers		Binge drinkers	
	Frequency	Intensity	Frequency	Intensity
Linear time trend	−0.06*	−0.03	−0.18*	−0.03
	(0.03)	(0.03)	(0.10)	(0.03)
Alcohol duty change 08	−0.01	−0.02	−0.12	−0.08
	(0.03)	(0.03)	(0.22)	(0.06)
INCOME1 (reference)				
INCOME2	0.08	0.07	0.64	0.13
	(0.07)	(0.06)	(0.44)	(0.12)
INCOME3	0.23	0.17	0.94	0.22
	(0.14)	(0.12)	(0.64)	(0.18)
INCOME4	0.34	0.21	1.46	0.31
	(0.22)	(0.19)	(0.95)	(0.26)
INCOME5	0.52	0.29	2.12	0.43
	(0.32)	(0.28)	(1.33)	(0.37)
INCOME6	0.60*	0.25	2.22	0.33
	(0.32)	(0.28)	(1.35)	(0.38)
COMPULSORY SCHOOL (reference)				
COLLEGE	0.08	−0.12*	0.44	−0.21**
	(0.07)	(0.06)	(0.33)	(0.09)
UNIVERSITY	−0.02	−0.61***	−4.38	−1.42*
	(0.10)	(0.09)	(2.75)	(0.77)
LNAGE	0.10***	0.00	−0.80	−0.16
	(0.03)	(0.03)	(0.60)	(0.17)
EMP (reference)				
INACTIVE	0.16**	0.18***	0.45	0.19**
	(0.07)	(0.05)	(0.35)	(0.09)
UNEMP	0.43*	0.18	0.75	0.07
	(0.24)	(0.21)	(0.50)	(0.14)
STUDENT	0.06	−0.03	−0.72*	−0.26**
	(0.05)	(0.05)	(0.43)	(0.12)
COHABIT	−0.36	−0.25	−1.28*	−0.31
	(0.24)	(0.21)	(0.74)	(0.21)
IMR	1.37	0.88	5.54	1.21
	(1.02)	(0.89)	(3.38)	−0.95
CONSTANT	−0.83	2.20***	7.60*	4.52***
	(0.81)	(0.70)	(4.36)	(1.22)
Participation observations	67,601	67,601	67,601	67,601
Freq/intens observations	17,738	17,738	6061	6061
Proportion drink/binge	26 %	26 %	9 %	9 %

Results are conditional on spirit drinking participation. Reference categories are income <10,000 sek, compulsory schooling, employed, and living alone. Alcohol duty change is a dummy set to one after January 1, 2008, when there was a change in beer and wine excise duties. Month dummies to capture resampling effects and seasonality and regional dummies are included in all models (reference categories: Stockholm and January). Testing the null of no effect: *** p < 0.01; ** p < 0.05; * p < 0.1

**Table 13:** Frequency and intensity demand equation estimates, females (price indices results)

	All drinkers		Binge drinkers	
	Frequency	Intensity	Frequency	Intensity
Beer				
Linear time trend	−0.11	0.05	−0.13	0.13
	(0.07)	(0.03)	(0.08)	(0.09)
Ln (beer price index)	−1.09	0.59	−0.38	0.01
	(1.17)	(0.53)	(1.21)	(1.28)
Wine				
Linear time trend	−0.01***	−0.01***	−0.02	0.00
	(0.00)	(0.00)	(0.01)	(0.01)
Ln (wine price index)	−0.59**	0.02	1.98	−2.88*
	(0.30)	(0.18)	(1.70)	(1.74)
Spirits				
Linear time trend	−0.08**	−0.04	−0.13	−0.04*
	(0.04)	(0.03)	(0.08)	(0.02)
Ln (spirit price index)	−0.76	−0.14	6.02	1.03
	(0.65)	(0.46)	(5.71)	(1.53)

See notes for Table 10. For each alcohol type the results presented are from separately estimated regressions. Results for income, education, employment status, and cohabiting are not shown but are included in the regression. The excise duty rate change in January 2008 is not controlled for in these results

**Table 14:** Alcohol log–log demand equation estimates conditional on participation, males

Variables	Pure alcohol consumed by all drinkers			Pure alcohol consumed by binge drinkers		
	Beer	Wine	Spirits	Beer	Wine	Spirits
Alcohol duty change 08	−0.01	0.01	−0.05	0.01	0.04	−0.37**
	(0.03)	(0.03)	(0.03)	(0.03)	(0.05)	(0.17)
Linear time trend	−0.01*	−0.03***	−0.04**	−0.01	0.03	−0.14***
	(0.01)	(0.01)	(0.02)	(0.01)	(0.02)	(0.05)
INCOME1 (reference)						
INCOME2	0.07	0.05	−0.06	0.06	−0.30**	0.55*
	(0.06)	(0.05)	(0.09)	(0.07)	(0.13)	(0.30)
INCOME3	0.20**	0.32***	0.07	0.14	−0.54**	1.12**
	(0.10)	(0.07)	(0.16)	(0.10)	(0.23)	(0.46)
INCOME4	0.36**	0.56***	0.17	0.23	−0.84**	1.86***
	(0.14)	(0.10)	(0.24)	(0.16)	(0.38)	(0.70)
INCOME5	0.39**	0.86***	0.24	0.20	−0.95*	2.41***
	(0.16)	(0.13)	(0.31)	(0.19)	(0.49)	(0.89)
INCOME6	0.32*	1.11***	0.26	0.10	−0.79	2.47***
	(0.16)	(0.14)	(0.33)	(0.19)	(0.51)	(0.91)
COMPULSORY SCHOOL (reference)						
COLLEGE	0.06	0.37***	−0.10**	0.04	−0.23	0.36
	(0.04)	(0.05)	(0.05)	(0.06)	(0.19)	(0.23)
UNIVERSITY	−0.16***	0.65***	−0.29***	−0.17***	−0.06	−0.28**
	(0.02)	(0.08)	(0.05)	(0.02)	(0.20)	(0.11)
LNAGE	−0.98***	0.91***	−0.17***	−0.79***	1.48***	−3.28***
	(0.10)	(0.07)	(0.05)	(0.29)	(0.27)	(1.17)
EMP (reference)						
INACTIVE	−0.29***	0.00	0.19***	−0.25*	0.62**	−0.84*
	(0.06)	(0.02)	(0.03)	(0.15)	(0.25)	(0.46)
UNEMP	0.13***	0.16***	0.22***	0.12**	−0.02	0.23
	(0.04)	(0.05)	(0.05)	(0.05)	(0.09)	(0.23)
STUDENT	−0.12***	0.38***	0.07	−0.11**	−0.09	−0.26
	(0.04)	(0.07)	(0.08)	(0.05)	(0.11)	(0.22)
COHABIT	−0.22***	0.21***	−0.16***	−0.22***	0.10***	−0.57***
	(0.02)	(0.03)	(0.03)	(0.04)	(0.03)	(0.16)
CONSTANT	6.11***	−2.40***	2.82***	5.83***	−0.01	8.05***
	(0.11)	(0.49)	(0.48)	(0.58)	(0.41)	(1.93)
IMR	0.39	0.98***	0.32	0.27	−1.94**	5.70***
	(0.32)	(0.21)	(0.69)	(0.42)	(0.90)	(1.98)
Participation observations	59,251	59,251	59,251	59,251	59,251	59,251
Quantity observations	35,650	31,925	31,244	19,297	14,414	16,641
Proportion drink/binge	60 %	54 %	53 %	33 %	24 %	28 %

Results are conditional on beer/wine/spirit drinking participation. Reference categories are income <10,000 sek, compulsory schooling, employed, and living alone. Alcohol duty change is a dummy set to one after January 1, 2008, when there was a change in beer and wine excise duties. Month dummies to capture resampling effects and seasonality and regional dummies are included in all models (reference categories: Stockholm and January). Testing the null of no effect: *** p < 0.01; ** p < 0.05; * p < 0.1

**Table 15:** Alcohol log–log demand equation estimates conditional on participation, males (price indices results)

Variables	Pure alcohol consumed by all drinkers			Pure alcohol consumed by binge drinkers		
	Beer	Wine	Spirits	Beer	Wine	Spirits
Linear time trend	−0.01**	−0.04***	−0.05***	−0.00	0.01	−0.18***
	(0.00)	(0.01)	(0.02)	(0.01)	(0.02)	(0.06)
ln (beer price index)	−0.53**			−0.56*		
	(0.24)			(0.29)		
ln (wine price index)		−0.81*			−3.14***	
		(0.48)			(1.12)	
ln (spirit price index)			−0.36			3.15
			(0.53)			(3.06)

See notes for Table 14. Results for income, education, employment status, and cohabiting are not shown but are included in the regression. The excise duty rate change in January 2008 is not controlled for in these results

**Table 16:** Alcohol log–log demand equation estimates conditional on participation, females

Variables	Pure alcohol consumed by all drinkers			Pure alcohol consumed by binge drinkers		
	Beer	Wine	Spirits	Beer	Wine	Spirits
Alcohol duty change 08	−0.06	0.00	−0.02	0.08	−0.01	−0.20
	(0.07)	(0.02)	(0.05)	(0.10)	(0.04)	(0.27)
Linear time trend	−0.05*	−0.02***	−0.10*	−0.01	−0.01	−0.21*
	(0.03)	(0.01)	(0.05)	(0.04)	(0.02)	(0.12)
INCOME1 (reference)						
INCOME2	0.27**	0.12***	0.15	0.08	0.11	0.76
	(0.12)	(0.04)	(0.11)	(0.21)	(0.13)	(0.54)
INCOME3	0.50*	0.30***	0.39*	−0.01	0.09	1.15
	(0.27)	(0.06)	(0.23)	(0.28)	(0.19)	(0.77)
INCOME4	0.68*	0.48***	0.56	0.00	0.17	1.77
	(0.37)	(0.09)	(0.35)	(0.43)	(0.29)	(1.16)
INCOME5	0.77*	0.67***	0.81	−0.16	0.29	2.55
	(0.44)	(0.11)	(0.52)	(0.55)	(0.39)	(1.62)
INCOME6	0.85*	0.74***	0.85	−0.17	0.40	2.55
	(0.51)	(0.12)	(0.52)	(0.60)	(0.43)	(1.64)
COMPULSORY SCHOOL (reference)						
COLLEGE	0.31*	0.26***	0.04	−0.07	0.19	0.74
	(0.19)	(0.05)	(0.07)	(0.29)	(0.26)	(0.60)
UNIVERSITY	0.09	0.36***	−0.04	−0.12	0.32*	0.24
	(0.13)	(0.07)	(0.11)	(0.13)	(0.18)	(0.40)
LNAGE	−1.26***	0.43***	−0.63***	−0.53	0.57	−5.80*
	(0.25)	(0.07)	(0.17)	(1.18)	(0.72)	(3.35)
EMP (reference)						
INACTIVE	−0.71*	−0.12***	0.10*	0.25	0.00	−0.97
	(0.43)	(0.03)	(0.06)	(0.58)	(0.27)	(0.73)
UNEMP	0.25***	0.04	0.34***	0.24*	0.00	0.64
	(0.09)	(0.04)	(0.11)	(0.13)	(0.06)	(0.43)
STUDENT	0.26*	0.30***	0.61	−0.09	0.11	0.82
	(0.14)	(0.06)	(0.38)	(0.18)	(0.13)	(0.61)
COHABIT	−0.07	0.12***	0.03	−0.09	−0.03	−0.98*
	(0.06)	(0.02)	(0.09)	(0.25)	(0.15)	(0.52)
CONSTANT	3.33***	0.16	1.37	4.84***	0.74	12.11**
	(1.20)	(0.48)	(1.31)	(1.50)	(0.88)	(5.31)
IMR	2.35	0.56**	2.25	−0.37	−0.02	6.75
	(1.55)	(0.26)	(1.65)	(1.65)	(1.14)	(4.11)
Participation observations	67,601	67,601	67,601	67,601	67,601	67,601
Quantity observations	17,148	42,208	17,738	6117	9114	6061
Proportion drink/binge	25 %	62 %	26 %	9 %	13 %	9 %

Results are conditional on beer/wine/spirit drinking participation. Reference categories are income <10,000 sek, compulsory schooling, employed, and living alone. Alcohol duty change is a dummy set to one after January 1st 2008 when there was a change in beer and wine excise duties. Month dummies to capture resampling effects and seasonality and regional dummies are included in all models (reference categories: Stockholm and January). Testing the null of no effect: *** p < 0.01; ** p < 0.05; * p < 0.1

**Table 17:** Alcohol log–log demand equation estimates conditional on participation, females (price indices results)

Variables	Pure alcohol consumed by all drinkers			Pure alcohol consumed by binge drinkers		
	Beer	Wine	Spirits	Beer	Wine	Spirits
Linear time trend	−0.06*	−0.03***	−0.12*	−0.00	−0.02	−0.17*
	(0.04)	(0.00)	(0.06)	(0.05)	(0.01)	(0.10)
ln (beer price index)	−0.50			−0.37		
	(0.64)			(0.56)		
ln (wine price index)		−0.58			−0.90	
		(0.38)			(1.74)	
ln (spirit price index)			−0.90			7.05
			(1.04)			(7.04)

See notes for Table 16. Results for income, education, employment status, and cohabiting are not shown but are included in the regression. The excise duty rate change in January 2008 is not controlled for in these results

## Appendix 2

### Missing data

The data for the years 2004 through to 2011 consists of a total of 144,025 observations. The final sample size is 126,852 after accounting for missing data (see Table 18).

**Table 18:** Summary of missing data by variable

Variable	% Missing (%)	*N* missing
DRINKER	0.70	1013
DRINK_BEER	2.99	4311
DRINK_WINE	3.01	4329
DRINK_SPIRIT	3.27	4709
BINGE DRINKER	2.77	3983
INCOME1	6.27	9035
COMPULSORY SCHOOL	0.00	1
LNAGE	0.00	1
MALE	0.00	1
COHABIT	0.02	32
EMP	2.12	3047
*N*		144,025

*Source*: Monitor project data years 2004–2011

Linear regression analysis is used to assess potential non-response bias (see Table 19). Missing values regarding alcohol (this is a combined variable which records missing if the respondent is missing data for whether they are a drinker, whether they drank beer, wine or spirits or whether they are a binge drinker) are predicted by younger respondents, with lower income and who are male with all other variables seemingly unimportant (We define important as both statistically significant at the 1 % level and a coefficient effect size of at least 1 %). Of the explanatory variables, our missing observation analysis finds that interpretation of income correlations will be for a subpopulation that is possibly more educated, male, younger, and more likely to be employed and employment status correlations will be for a subpopulation that possibly has higher income but lower levels of education.

**Table 19:** Regression analysis results of missing data

Missing = 1	alc_missing	INCOME_missing	UNEMP_missing
INCOME2	−0.005**		0.007***
	(0.002)		(0.002)
INCOME3	−0.003		−0.007***
	(0.002)		(0.002)
INCOME4	−0.011***		−0.016***
	(0.002)		(0.002)
INCOME5	−0.017***		−0.017***
	(0.003)		(0.002)
INCOME6	−0.016***		−0.019***
	(0.003)		(0.002)
GYMNASIET	−0.008***	−0.019***	0.009***
	(0.002)	(0.002)	(0.001)
UNIVERSITET	−0.007***	−0.025***	0.013***
	(0.002)	(0.002)	(0.001)
LNAGE	−0.013***	0.017***	−0.010***
	(0.002)	(0.003)	(0.001)
MALE	0.012***	−0.015***	−0.010***
	(0.001)	(0.001)	(0.001)
INACTIVE	−0.008***	0.033***	
	(0.002)	(0.002)	
UNEMP	0.007*	0.049***	
	(0.004)	(0.005)	
STUDENT	−0.003	0.028***	
	(0.003)	(0.003)	
COHABIT	−0.006***	0.000	0.005***
	(0.001)	(0.001)	(0.001)
Observations	132,239	140,949	134,973
*R*-squared	0.028	0.012	0.007

Monitor project data years 2004–2011. Month dummies to capture resampling effects and seasonality and regional dummies are included in all models (reference categories: Stockholm and January). Robust standard errors are reported in* parentheses*. Testing the null of no effect: *** p < 0.01; ** p < 0.05; * p < 0.1

## Footnotes

1 Translating the Monitor project binge drinking threshold into UK alcohol units: one bottle of wine (75 cl) (assume 12 % vol =9 UK units), five shots of spirit (25 ml) (assume 40 % vol = 5 UK units), four cans of strong beer/cider (≥3.5 %) (assume 5 % vol 330 ml cans, =6.6 UK units) or six cans of low alcohol content beer (3.5 %) (assume 3.5 % vol 330 ml cans, =6.9 UK units). The Audit questionnaire defines a binge drinker as someone drinking six or more units if female or eight or more units if male.

2 Individuals with missing values are dropped from the dataset.

3 These additional estimates include a linear time trend and all other controls but not the excise rate dummy variable so that interpretation is clear and we only use own prices due to the high correlation of the price indices. Models have been estimated using all price indices in all models (results not shown) but because of the high correlation between the indices, the results were difficult to interpret. There appears to be not enough variation between the price indices over time to disentangle own and cross price elasticities during this time period. We therefore only model using our own price elasticities.

4 The log–log model of demand is limiting in that the model requires the elasticities to be unitary otherwise the expenditures will not be equal to total outlay (i.e., the budget will not add up) unless analysis is within a restricted range of total outlay [15]. However, less restrictive models require data on budget shares, which are not available. In part, we get around this issue as a consequence of the analysis considering both all drinkers *and* binge drinkers.
